# Effect of Strain Rate on the Formability Prediction of Cold-Rolled DX56D+Z100-M-C-O Steel Sheets

**DOI:** 10.3390/ma19010099

**Published:** 2025-12-27

**Authors:** Vít Novák, František Tatíček, Ondřej Stejskal, Tomasz Trzepieciński, Krzysztof Żaba

**Affiliations:** 1 Department of Manufacturing Technology, Faculty of Mechanical Engineering, Czech Technical University in Prague, Technická 4, 166 07 Prague, Czech Republic; frantisek.taticek@fs.cvut.cz (F.T.); ondrej.stejskal@fs.cvut.cz (O.S.); 2 Department of Manufacturing Processes and Production Engineering, Faculty of Mechanical Engineering and Aeronautics, Rzeszow University of Technology, al. Powst. Warszawy 8, 35-029 Rzeszów, Poland; tomtrz@prz.edu.pl; 3 Department of Metal Working and Physical Metallurgy of Non-Ferrous Metals, Faculty of Non-Ferrous Metals, AGH University of Krakow, al. Adama Mickiewicza 30, 30-059 Cracow, Poland; krzyzaba@agh.edu.pl

**Keywords:** formability, forming limit diagram, steel sheet, tensile test, mechanical properties, sheet metal forming

## Abstract

Formability testing is a fundamental method for determining sheet metal’s susceptibility to deep drawing operations. This article presents the results of formability analysis of several batches of 0.7 mm thick cold-rolled DX56D+Z100-M-C-O steel sheets. As part of the preliminary tests, mechanical properties of the tested steel sheets were determined. The ARAMIS digital image correlation system was used to determine the formability of sheet metal during the hemispherical punch stretching test. The stretching tests were conducted over a wide range of strain rate variations between 2 mm/min and 17 mm/min. A total of 540 individual geometry measurements were taken to analyze the test material’s formability. It was observed that with increasing strain rate, the strength properties increased, while the plastic properties decreased. From the perspective of formability, the margin of plasticity (the ratio of yield strength to tensile strength) deteriorated with increasing strain rate in tensile tests. Forming limit curves revealed that at higher strain rates, the metal sheet’s formability decreased. A reduction in the safety margins with an increasing hemispherical punch stretching test speed was also observed.

## 1. Introduction

Deep drawing processes are one of the most popular sheet metal-forming techniques. These methods can be used to form complex-shaped drawpieces, particularly in the automotive industry. A properly designed sheet metal-forming process requires determining potential areas of wrinkling, springback, material failure, and the strain limits [1,2]. Deep-drawn sheets typically exhibit anisotropy in their plastic properties, which makes it difficult to accurately assess the forming performance of sheet metals when forming drawpieces with complex shapes [3]. Testing sheet metal properties involves conducting numerous experimental tests, the most common of which are uniaxial and biaxial tensile tests, formability tests, and hardness measurements [4]. Friction tests [5], tribological analysis of coatings [6], and surface topography measurements [7] are commonly performed during the design phase of sheet metal-forming technology.

The simulation programs that predict the formability of sheet metals, which in turn influence the input conditions, are widely used in deep drawing technology [7,8]. Computational models are also being developed that take into account the change in microstructure behavior with changing deformation conditions [9,10]. When using the simulation program, it is necessary to adequately describe the material behavior [11,12]. Basic mechanical parameters and the forming limit curve (FLC) describe the material’s behavior in complex deformation states [13]. The FLC shows the boundary at which material failure occurs due to major and minor deformations. It is divided into the safe deformation area and the area where material failure occurs. In practice, the complex drawpieces in which the individual sections undergo deformation in different stress states and different deformation rates are most often formed. These parameters have a significant influence on the metal sheets’ formability at the deep drawing process. With the development of metal-forming machines and advanced materials, the material cards need to be updated and closer to the real working conditions. To ensure the stability of the forming process, the highest possible level of material usability, and the lowest possible level of rejected parts, it is necessary to properly identify the specific material being processed [14,15,16,17].

Many methods have been developed to predict the sheet metal’s drawability, including the Erichsen/Olsen cupping test, hydraulic bulge test, uniaxial tensile test, Nakajima test (hemispherical cupping die), and Marciniak test (flat die) [18,19]. These methods help identify areas of potential weakness or failure of the material. These methods differ primarily in sample geometry, tool geometry, and process parameters. The Erichsen test primarily measures stretch formability using a hemispherical punch to deform the sheet metal until fracture [20]. This test is a robust quality control method used to assess the adhesion of coatings and ductility of sheet metals [21]. In the hydraulic bulge test, the load is applied by a fluid, which eliminates the frictional forces that can occur between the punch and the sheet metal. One of the main advantages of the bulge test is the possibility of reaching high levels of deformation before fracture [22]. The Nakajima test allows for a wider range of strain paths, from uniaxial to biaxial tension, by varying the sample geometry. The Nakajima test can be adapted for various thicknesses. It can be combined with the digital image correlation (DIC) technique to provide accurate strain measurements and track crack locations [23,24].

Forming limit diagrams (FLDs) are one of the most useful tools for assessing the formability of sheet materials, as indicated by Keeler [25] and Goodwin [26]. FLDs are determined under various forming conditions according to in-plane [27] or out-of-plane strategies [28]. In addition to experimental methods for determining FLDs, which are time-consuming, theoretical or numerical methods have been developed over the years. The approach proposed in [29] allows for the design of sheet-forming processes without the need for experimental studies using real forming tools. The system uses a finite element-based program, which enables the prediction of forming forces, strain and stress distribution, and the determination of the material state based on FLD. Models based on bifurcation theory, including Swift and Hill approaches, are not applicable to complex forming conditions [30]. The accuracy of these models can be improved by considering parameters of the tested sheet metal, including void growth and surface roughness [31,32]. Continuum damage mechanics-based models are the most complex and take into account parameters such as dislocation density, work hardening, and void growth [33]. DiCecco et al. [34] proposed a physically based enhanced curvature method that detects necking based on the onset of local necking curvature. Time-dependent methods, including the statistics-based, strain rate-based, and mechanism-based strain gradient plasticity methods, are attempted to detect an acute neck by tracking the history of either the thinning strain rate or the major strain rate on the sheet surface.

In the automotive industry there are increasing demands for high productivity, but also for safety, environmental responsibility, and sustainability of forming processes [35,36]. The vast majority of passenger vehicles are manufactured from low-carbon steel sheets or aluminum alloys, with stainless steel sheets and titanium alloys used to a lesser extent [37]. Due to their mechanical properties, cold-rolled steel sheets are suitable for the production of drawpieces requiring a smooth surface and precise forming, especially of exterior and interior car body panels. Due to the manufacturing process (rolling), sheet metals are characterized by anisotropy of mechanical properties, which must be considered when designing the forming technology [38]. The sheet metals are supplied by various manufacturers in the form of strips. Each strip represents a batch of material that is uniquely identified. There is some variation in properties between suppliers, but also between batches of material, which affects formability [15,39]. The car body parts are required to be precise enough, and the drawpieces must be free of defects (cracks, wrinkles, scratches). The material properties have a major influence on the stability of the forming process [40,41,42]. Therefore, this paper, for the first time, aims to compare the mechanical properties of various batches of cold-rolled DX56D+Z100-M-C-O steel sheets determined in the uniaxial tensile test and Nakazima formability test, considering different deformation conditions. This material is currently one of the most used for automotive body construction, both in terms of thickness and quality. The material was selected to allow obtaining test samples from various suppliers while also monitoring the variation in properties across different heats from the chosen suppliers. A thickness of 0.7 mm was chosen to ensure that there are multiple potential suppliers for one material grade. Samples were cut in three directions in respect to the sheet-rolling direction. Batches from different manufacturers and the position of the samples in the coil were also considered. The effect of chemical composition of specific batches of DX56D+Z100-M-C-O steel on basic mechanical properties was analyzed and discussed. Moreover, Nakazima tests combined with the DIC technique were used to study the effect of different chemical compositions of DX56D+Z100-M-C-O steel sheet batches on the formability of sheet metals under various strain rates.

## 2. Material and Methods

### 2.1. Material

**Figure 1:**
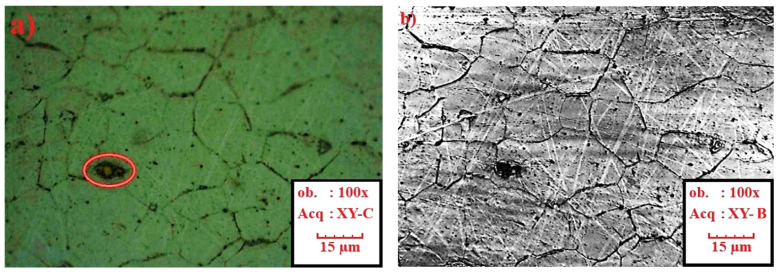
Microstructure of DX56D+Z100-M-C-O with visible TiCN inclusions on (**a**) a color optical 2D image and (**b**) a confocal image. The red circle marks a TiCN (titanium carbonitride) inclusion, which forms through the reaction of titanium with carbon and nitrogen.

Formability tests were carried out for 0.7 mm thick DX56D+Z100-M-C-O steel sheet (equivalent of DX56D+Z100-M-C-O steel sheet according to EN 10346:2015 [43]). DX56D+Z100-M-C-O is an interstitial-free mild steel grade with super deep drawing capabilities, intended for use in demanding stamping applications. It is a noble low-carbon ferritic steel microalloyed with titanium (the standard prescribes a maximum amount of 0.3 wt.%), which serves as a carbonitriding stabilizer to completely clean ferrite from interstitially dissolved C and N. Reducing the content of these elements increases the value of normal anisotropy and guarantees better deep drawing properties. Titanium forms TiCN inclusions, which can be seen as sharp yellow edges in the material (Figure 1) under microscopic observation on an Olympus LEXT OLS 3000 microscope (Olympus Corporation, Tokyo, Japan). The technical requirements for DX56D+Z100-M-C-O steel (Table 1) are listed in the VDA 239-100 standard [44]. Requirements for the chemical composition of DX56D+Z100-M-C-O steel in accordance with the VDA 239-100 standard [44] are shown in Table 2. The tested sheets were supplied by two different manufacturers (batches A and B from Salzgitter, Lower Saxony, Germany and batches C–E from U. S. Steel Košice, s.r.o.; Šaca, Košice, Slovakia). To achieve the widest possible range of results, various suppliers of this material grade were selected, and different heats from the chosen suppliers were analyzed. The objective was to monitor not only the influence of the supplier on the available forming capacity but also the effect of individual heats on the scatter of results.

### 2.2. Tensile Test

**Figure 2:**
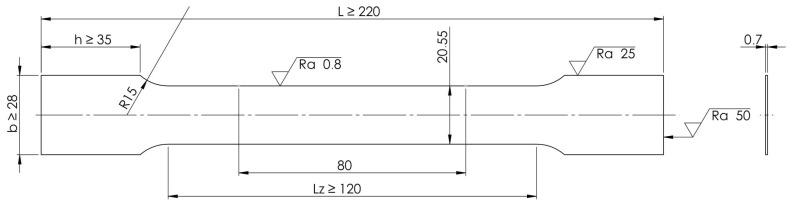
Shape and dimensions of the samples used for uniaxial tensile tests (Unit: mm).

A tensile test was performed at ambient temperature (20 °C) on a universal electromechanical testing machine of the LabTest model 5.100SP1 (LABORTECH s.r.o.; Opava, Czech Republic) series according to the test standard EN ISO 6892-1:2020 [45]. The LE05 extensometer (LABORTECH s.r.o.; Opava, Czech Republic) was used for strain measurement. Figure 2 shows the geometry of samples used in the uniaxial tensile test. In this study, over 540 material samples from 5 batches (A–E) underwent tensile testing in three principal directions at crosshead feed rates ranging from 2 to 600 mm/min. Tensile test specimens were prepared using electrical discharge machining and cut from the sheet in the rolling direction (RD), transverse direction (TD), and at an angle of 45° relative to RD. The following parameters were determined: elongation A_80_, yield strength R_p0.2_, ultimate tensile strength R_m_, and plastic elongation A_g_ measured at maximum load.

### 2.3. Digital Image Correlation

**Figure 3:**
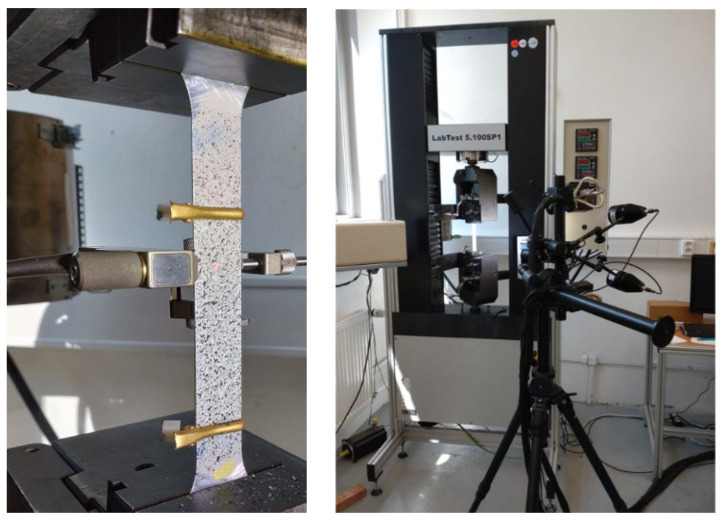
Specimen prepared and clamped for tensile testing; measurement performed using the ARAMIS DIC system.

The FLCs were determined in accordance with the EN ISO 12004-2:2021 [28] standard. This standard prescribes the measurement of at least five different geometries to achieve different ratios of major and minor deformation. Individual changes in the ratio were achieved by a different width of the sample (20, 65, 105, 120, and 140 mm). Individual samples were obtained from the electroerosive machined sheet metal strips. Subsequently, the samples were degreased, rinsed, and dried. The ARAMIS high-speed DIC system (Braunschweig, Germany) was used to analyze the deformation state of workpieces. Two charge-coupled device (CCD) cameras at a defined distance from each other observed the samples shown in Figure 3. A change in deformation occurs over time, which was recorded and then compared. There are two correlations: stereo correlation comparison and temporal comparison [46].

A precondition for correct measurements was the calibration of the system and its stabilization during the measurement. This method provides valuable information during the formability test, but due to the character of the pattern, it cannot be applied during measurements of drawpieces. The surface to be analyzed must be visible throughout the analysis. The sample surface was covered with a random pattern that allows for scanning. The scanner measures each individual point and monitors its relative motion during deformation. Prior to applying the pattern, the sheet metal surface was thoroughly degreased.

Monitoring multiple points in a specific environment eliminates the risk of confusing individual tracked objects. These points are referred to as subsets, and the accuracy of the measurement is impacted by the size of the subsets and their overlap. The selection of appropriate settings yields the desired number of unique subsets, and the uniqueness of the subset neighborhood is determined by the quality of the surface being observed. The surface of workpieces must be non-repetitive, contrasting, and isotropic. Our approach involves generating a random spray pattern to establish these characteristics, but alternate methods are available [47].

**Figure 4:**
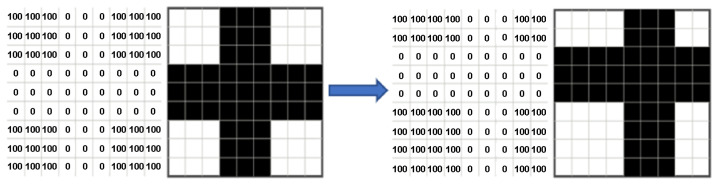
Example of grayscale in subset.

Numerical values are assigned on the grayscale to each subset of pixels. Afterward, the subsets are searched, and their relative motion is calculated when comparing each image (Figure 4). The standard procedure involves recording all possible subset locations and calculating a correlation function to obtain an optimum outcome. The least squares method was utilized by default [48].

**Figure 5:**
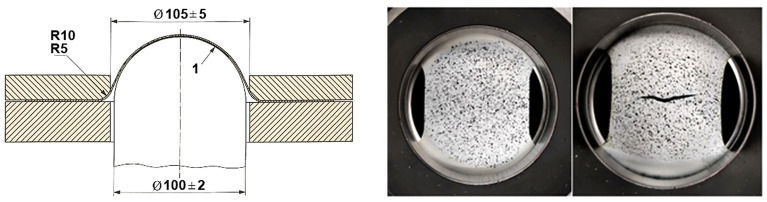
Scheme of the Nakajima test and the FLC sample before and after testing (Unit: mm).

**Figure 6:**
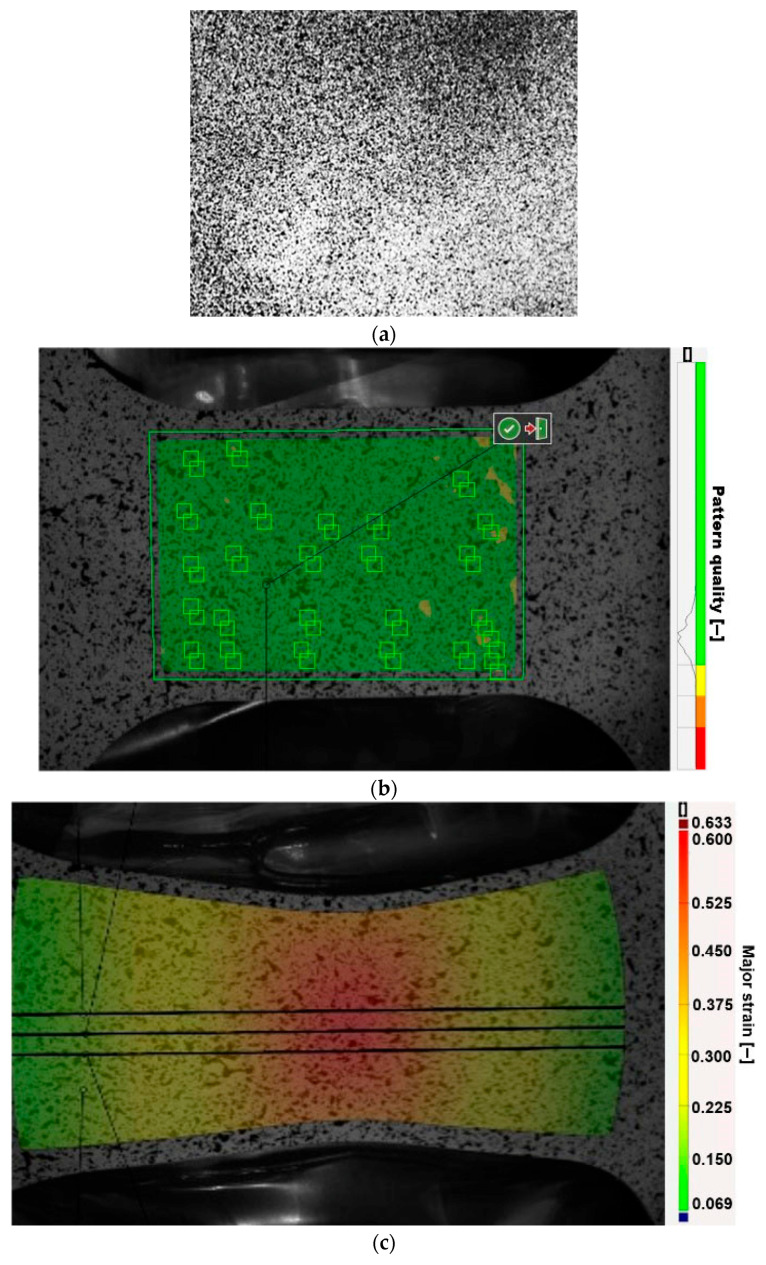
(**a**) reference spray, (**b**) spray quality verification, and (**c**) evaluation of deformations using the section method.

The samples were then placed into the BUP 600 (Zwick/Roell, Ulm, Germany) sheet metal testing machine, equipped with a hemispherical 100 mm diameter punch (Figure 5) and an ARAMIS non-contact measurement system. Before measurement with the ARAMIS system, a black and white coating is applied to the surface of the test object. This coating deforms in unison with the object’s surface. Figure 6a–c illustrate this process. During deformation, two CCD cameras capture images of the object based on the DIC principle. The measurement parameters were the loading speed between 2 mm/s and 17 mm/s, the sample orientation according to RD, and a blankholder force of 300 kN. By changing the loading speed to higher values, we follow the downward movement of the limit deformation curve and the position on the coil and the diffusion of mechanical properties in the coil. Samples were taken at three locations, including the start, center, and end of a 6 km long coil. Because according to the standard, a coil can be welded at two locations from production, the influence of the position of the samples on mechanical properties and formability was therefore investigated. The samples need to be sufficiently illuminated at higher speeds, because greater illumination of the sample is needed at a higher frame rate per second. To eliminate friction between the sample and the punch, the punch was equipped with a tribological system to reduce friction, which was unchanged during the measurement. For the test to be valid, a crack must occur at a distance corresponding to 15% (15 mm) of the punch diameter. This can only be achieved if friction at the interface between the punch and the tested material is minimized. The term “tribological system” refers internally to a combination of low-viscosity oils, PTFE (Teflon) tape, and PE tape.

**Figure 7:**
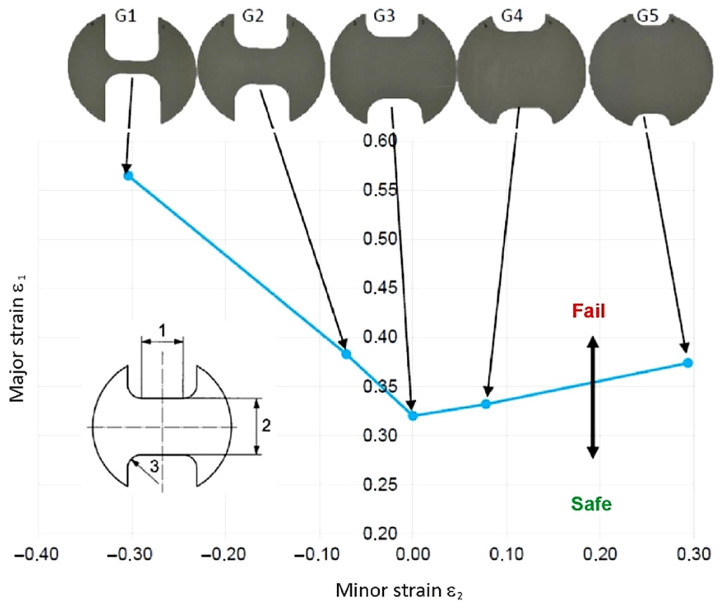
Method of creating a forming limit diagram.

The method of generating FLCs based on testing samples with different geometries is shown in Figure 7. For a punch diameter of 100 mm, the sample length (1 in Figure 7) was 50 mm. The width of the sample has values of G1 = 20, G2 = 65, G3 = 105, G4 = 120, and G5 = 140 mm (2 in Figure 7). The filled radius (3 in Figure 7) was 30 mm.

### 2.4. Analysis of Chemical Composition

**Figure 8:**
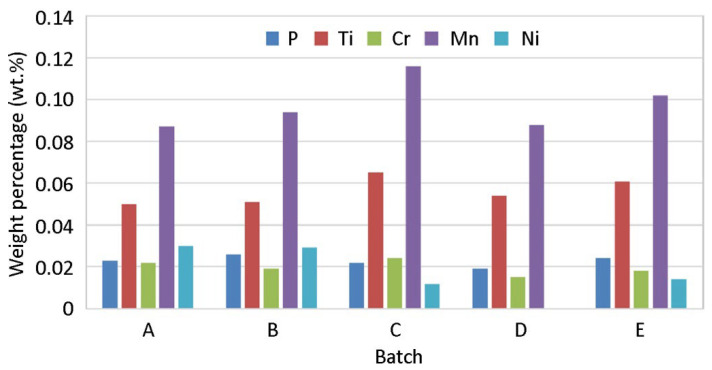
Chemical composition of different batches.

Spectral analysis was performed using a handheld Delta Professional device to determine the chemical composition of materials in individual batches. Prior to measurement, the zinc coating on the sheet surfaces was removed by rinsing in a 10% hydrochloric acid solution. According to Figure 8, approximately similar alloying element ratios appear in batches. The greatest difference between the tested batches occurs in the manganese content. For batch D, the absence of nickel can be seen, but a more thorough measurement would be needed to draw conclusions, as for the purposes of this experiment.

## 3. Results and Discussion

### 3.1. Tensile Tests

**Figure 9:**
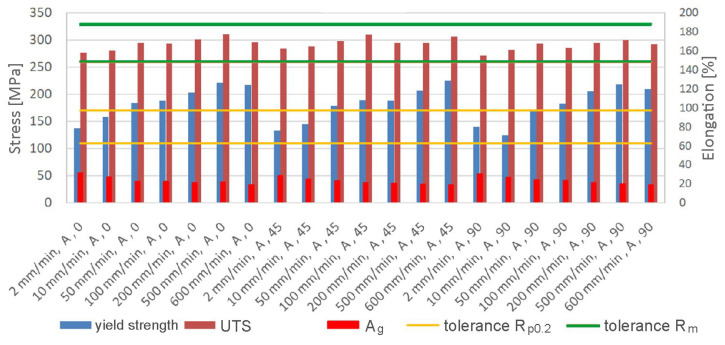
Effect of strain rate on mechanical properties of batch A.

This study analyzed multiple batches of material sourced from varied suppliers and labelled A through E. Distinct loading rates were adopted to attain different strain rates. Throughout this study, the analysis encompassed several parameters, including yield strength, ultimate tensile strength (UTS), elongation, strain-hardening exponent, and plastic anisotropy coefficient. Figure 9 illustrates the correlation between changes in strain rate and yield strength, ultimate tensile strength, and elongation. The UTS interval region, according to the standard, is visually represented in green, while the yield strength region is shown in yellow. The graph indicates that as the strain rate increases, the yield strength and UTS also increase, while the elongation decreases. It is apparent that the ratio of yield strength to ultimate tensile strength, an indicator of formability also known as the so-called ‘plasticity reserve’, increases as the strain rate increases. These observations are valid for all analyzed strip sample orientations. For the lowest analyzed strain rate (2 mm/min), yield strength varied between 135 MPa (0) and 142 MPa (45). For a strain rate of 600 mm/min, yield strength ranged between 208 MPa (90) and 223 MPa (45), representing an increase of over 50%. There is a tendency for UTS values to increase proportionally to strain rate; however, the higher the strain rate, the greater the R_p0.2_/R_m_ ratio. Increased yield strength and UTS, as a result of the work-hardening phenomenon, simultaneously reduces elongation A_g_. Increasing the strain rate from 2 mm/min to 600 mm/min resulted in a reduction in elongation between 33.6% (0) and 36.3% (90).

**Figure 10:**
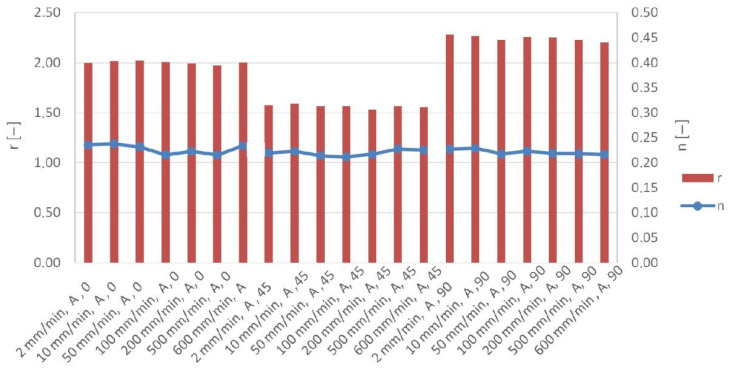
Effect of strain rate on coefficient of plane anisotropy r and strain-hardening exponent n for batch A.

There is a clear trend toward a decrease in the coefficient of anisotropy with strain rate. This decrease is most pronounced for the 90° orientation (Figure 10). For the orientation 0, the coefficient of anisotropy is only slightly sensitive to strain rate changes. For individual sample orientations, values of the coefficient of anisotropy are approximately 1.98–2.02 (0), 1.52–1.59 (45), and 2.18–2.25 (90). Large differences between the anisotropy coefficient values determined for different orientations are evident and can have a significant impact on the material’s behavior during further material processing. In processes such as deep drawing, knowledge of material anisotropy is crucial because it allows for predicting how the material will react in different orientations and preventing problems such as cracking. The strain-hardening exponent is only slightly sensitive to strain rate changes and ranges between 0.213 and 0.226 for all orientations and strain rates considered (Figure 10). A higher n-value indicates a more uniform distribution of strain throughout the material during deformation, which is desirable for processes like deep drawing.

**Figure 11:**
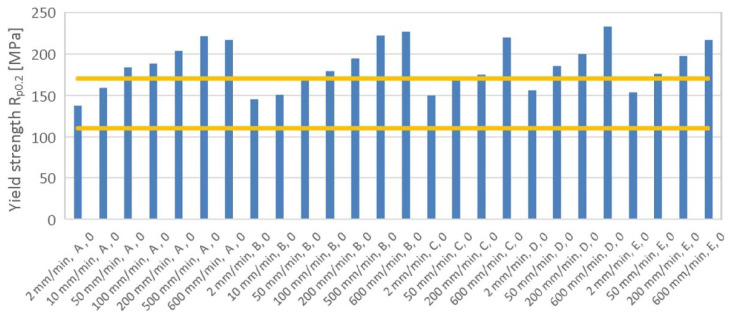
Effect of strain rate on yield strength for samples cut along the RD (all batches).

Figure 11 illustrates the effect of strain rate on yield strength for all analyzed material batches in the rolling direction. The graph displays the parameter interval values set by the standard. The results indicate that batch A has the lowest yield strength under standard conditions, while batches C, D, and E present the highest yield strength among all analyzed batches. It is noteworthy that all batches exhibit sensitivity to strain rate, with batch E being the least sensitive.

**Figure 12:**
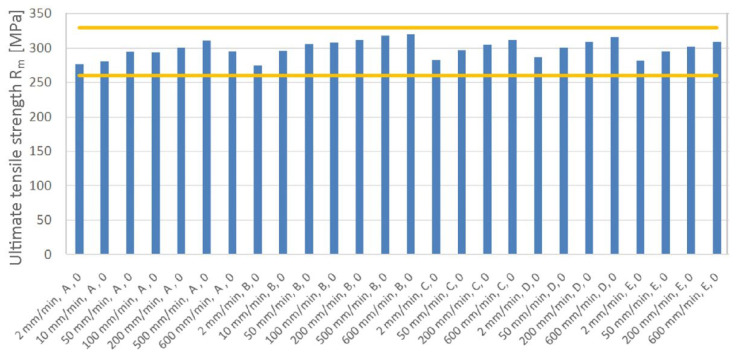
Effect of strain rate on ultimate tensile strength for samples cut along the RD (all batches).

For a strain rate of 2 mm/min, batch B displays the lowest ultimate strength values in the rolling direction (Figure 12). For all material batches analyzed, there is an increase in value of UTS as the strain rate increases; however, the increase is less intense than that for the yield strength. In terms of sensitivity to strain rate, batch E shows the lowest values. Taking into account all strain rates, the increase in strain rate from 2 mm/min to 600 mm/min resulted in an increase in UTS between 7.6% (batch A) and 12.5% (batch C).

**Figure 13:**
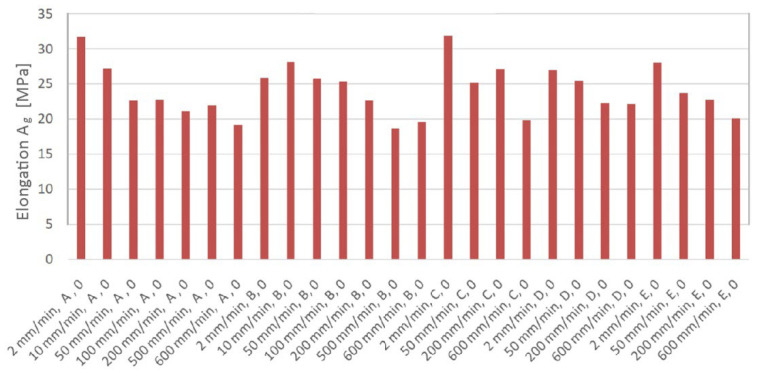
Effect of strain rate on elongation for samples cut along the rolling direction 0 (all batches).

The effect of strain rate on elongation is presented in Figure 13. Samples from batches A and C exhibit the highest similar elongation value at a strain rate of 2 mm/min. As strain rate increases, all batches show a tendency for elongation to decrease with increasing strain rate. Batch D was the least sensitive to the effect of strain rate. For this batch, in the strain rate range of 2–600 mm/min, elongation decreased by only 18.5%. In contrast, for batch A, increasing the strain rate from 2 to 600 m/min resulted in a reduction in elongation by approximately 40.6%.

**Figure 14:**
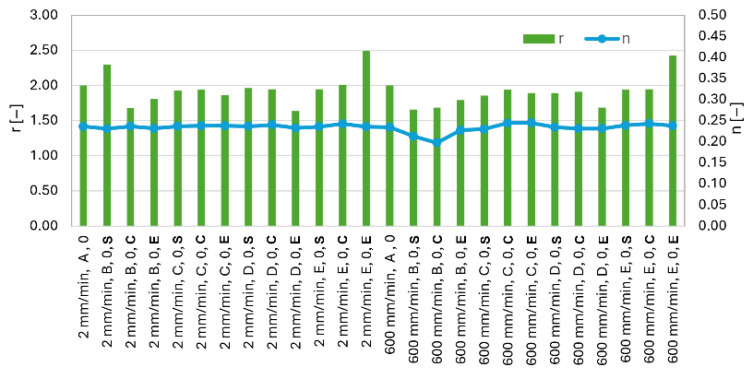
Effect of the strain-hardening exponent “n” and the plastic anisotropy coefficient “r” for different batches and positions within the coil.

When examining Figure 14, the values of the strain-hardening exponent and the plastic anisotropy coefficient indicate that the strain-hardening exponent remains consistent throughout the entire measurement and is not affected by the loading rate. The comparison chart was created for loading speeds of 2 mm/min and 600 mm/min to identify potential differences between the lowest and highest loading rates. Although slight variations in the plastic anisotropy coefficient can be observed, these are minor deviations in most cases and primarily occur between different batches. Noticeably higher coefficient values are observed, for example, in batch E at orientation at 0 and at the end position of the coil. The values are therefore increased for the same material, in the same orientation, and at the same position. It can thus be concluded that the loading rate does not have a significant effect on these parameters.

### 3.2. Formability Tests

To obtain a complete view of the measurement result, individual data was compared according to batch sizes and test speeds. Table 3 and Table 4 present the values of major (ε_1_) and minor (ε_2_) strains for samples G1–G4 (Figure 5) for selected batch B. Averaged data for individual positions on the roll (marked BS—start of coil, BC—center of coil, BE—end of coil) at test speeds of 2 mm/s (Table 3) and 17 mm/s (Table 4) are also presented.

**Figure 15:**
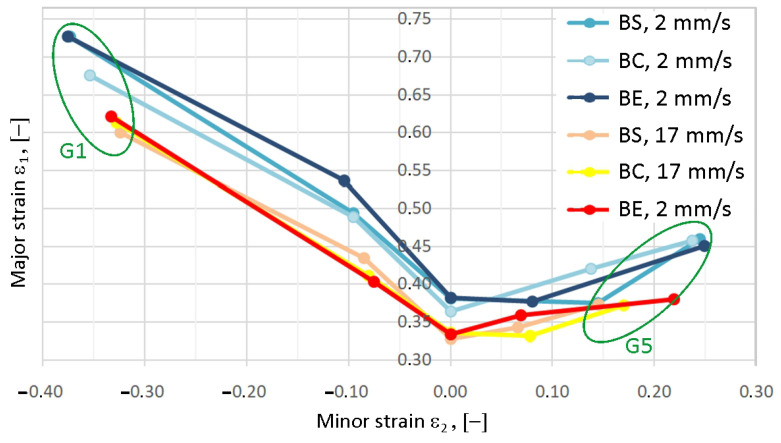
FLD for batch B; all types of samples; all test speeds.

**Figure 16:**
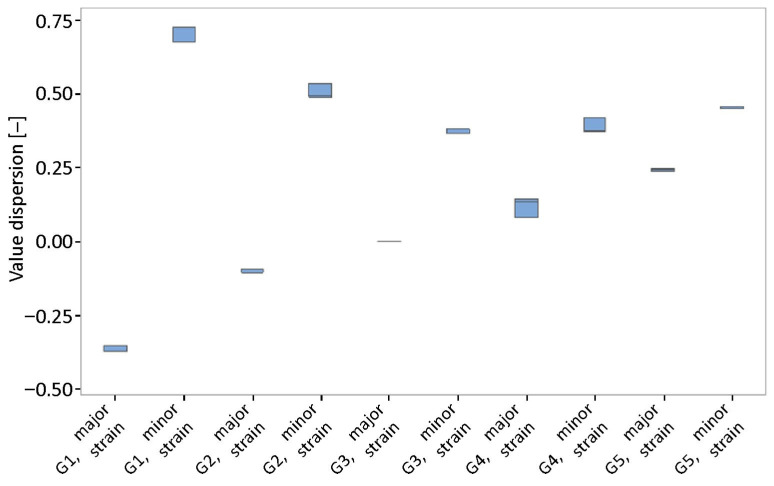
Dispersion of minor and major strains for individual geometries of samples G1–G5 (batch B).

Figure 15 shows the curves obtained by measuring at a test speed of 2 mm/s in shades of blue and the curves obtained at a test speed of 17 mm/s in light shades. Individual curves give us the limit of material failure at defined parameters of position and velocity. As the strain rate increases, the major strain values decrease. Simultaneously, the shift in extreme values (positive and negative) for samples G1 and G5 towards the ordinates indicates a decrease in the material’s plasticity. Points below the forming limit curve indicate successful forming, while points at or above the FLC indicate potential sheet cracking. As for the variability of formability, we can see that there are no major changes during the testing of batch B (Figure 16). However, the dispersion value of the minor strains is greater than that of the major strains.

**Figure 17:**
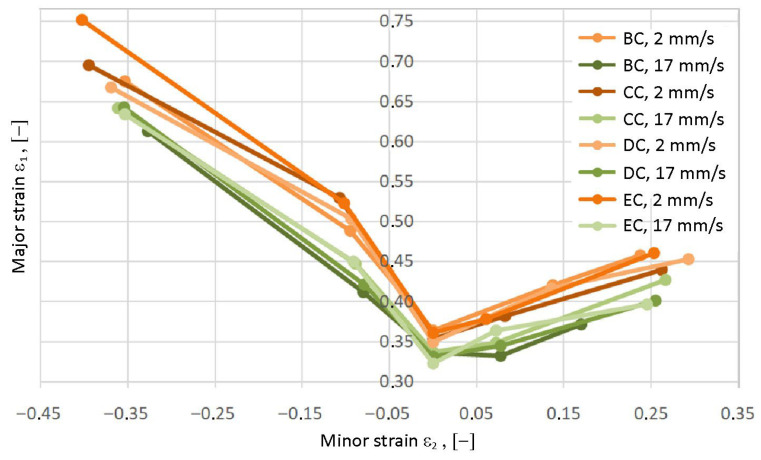
Forming limit curves for batches B–E (position: center of coil—BC, test speeds: 2 and 17 mm/s).

A comparison of the FLCs of samples from batches B-E is presented in Figure 17. The difference in individual curves is shown, and there is a division into two “groups of curves”, for higher and lower test speeds. The largest percentage difference (22.9%) in major strain values ε_1_ for the analyzed samples occurs for sample G1. In terms of additional minor strain values ε_2_ (samples G4–G5), the differences between major strain values for individual batches are significantly smaller than for samples G1 and G2. This indicates that under biaxial tension and stretch-forming conditions, the sheet material is less sensitive to strain rate compared to the simple tension state. In the groups of batches deformed at the same test speed, sample G3 showed the smallest differences in major strain ε_1_.

**Figure 18:**
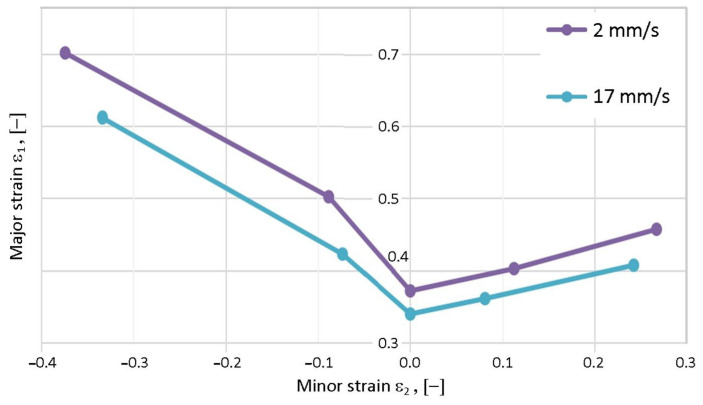
Average values of minor and major strains determined for test speeds of 2 and 17 mm/s (batches B–E).

Comparison of the average points of individual FLCs for test speeds of 2 and 17 mm/s, shown in Figure 18, is essential for obtaining a comprehensive analysis of the forming tool speed on formability. As strain rate increases, plasticity decreases more significantly under simple tension conditions compared to stretch forming. The reduction in major strains with increasing test speed under conditions with positive minor strains is approximately 8.75% (G4) and 10.5% (G5). In the range of negative minor strains, the reduction in major strains is between 12.8% (G1) and 16.0% (G2). Knowledge of the sheet metal’s formability limit allows modification of tool shape and contact conditions to ensure sheet metal deformation within the safe range.

**Figure 19:**
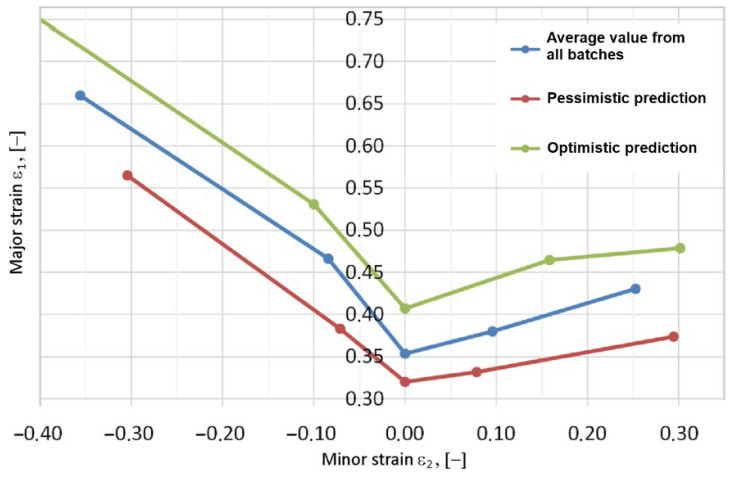
FLD for application in numerical simulations.

Figure 19 shows the margin for predicting the mechanical properties of the material, taking into account data for all batches of DX56D+Z100-M-C-O steel sheets. FLCs for different test speeds differ in position, and as the test speed of the material increases, the safe area decreases. For industrial applications, FLCs for the pessimistic variant are important because they ensure the stability of the forming process by ensuring the creation of forming strategies using the worst possible values of the workpiece material. At the same time, FLCs representing the optimistic variant (Figure 19) of the material correspond to the highest values of major strains determined experimentally. In the context of numerical modeling of sheet metal-forming processes using the finite element method, it is necessary to account for the dispersion of mechanical properties of sheets from different melts. Depending on the sample type (Figure 7), the difference in the margin of major strains ε_1_ between the optimistic and pessimistic variants can range from 20.9% to 28.32% (Figure 19). The difference between the points corresponding to minor strains for the pessimistic and optimistic variants can be as much as 26.2%.

Forming limit curves are an indispensable tool in the analysis and design of the sheet metal-forming processes. Their use in numerical models allows not only the prediction of crack occurrence but also the optimization of technological processes, which translates into material and time savings. Comparing the results of finite element-based simulations with the experimentally determined FLCs allows for the assessment of the quality of the material model, verification of the simulation’s reliability, and confirmation of the validity of the assumptions made.

The research results presented in this paper highlight the impact of variations in the mechanical properties of the test material, resulting from the requirements of relevant standards, on the basic mechanical properties of sheet metal. Designing the technological process for sheet metal forming should be tailored to the minimum plastic properties in the worst-case variant of FLC. Determining mechanical properties for a single melt may not guarantee adequate stability of mechanical parameters. Consequently, unfavorable phenomena may occur during the sheet-forming process, limiting the production of drawpieces with the desired dimensions or shapes [49]. Beyond their practical aspects, the research results presented in this paper are important for numerical modeling of sheet metal-forming processes. A proper material model is the foundation of reliable numerical simulation [50]. Taking into account appropriate sheet metal properties (e.g., anisotropy, work-hardening phenomenon, strain rate sensitivity) allows for accurate prediction of sheet metal behavior during the forming process, thus reducing pilot press tests and lowering industrial costs [51].

### 3.3. Metallographic Analysis

Metallographic observation was performed on the materials analyzed. Metallographic cuts were prepared for optical microscope observations. The samples’ surfaces were etched with a 2% Nital etchant to highlight the microstructure. Specimens were cut in the crack region from six FLC samples after the Nakajima test, representing three stress states for test speeds of 2 mm/s and 17 mm/s (Table 5). From the analyses carried out, it follows that with an increasing rate of deformation, the size of the deformation is localized to a smaller distance from the crack. Furthermore, deformation occurs mainly in the grains at a small depth below the surface. With increasing depth or distance from the crack, the amount of grain deformation decreases, indicating that the grains were less deformed. This is observed for all three stress states considered (uniaxial stress, plane stress, and biaxial stress).

## 4. Conclusions

In this study, over 500 material samples from five batches underwent tensile testing in three principal directions at crosshead feed rates ranging from 2 mm/min to 600 mm/min. Moreover, formability analysis was conducted using Nakazima tests supported by the DIC system ARAMIS. Based on the extensive research campaign, the following main conclusions were revealed:

- The yield strength and UTS of the test material increase with an increase in the strain rate. The increase is more pronounced in the yield strength than in the UTS. The ductility of the material determined by the elongation reduces with the increasing strain rate. However, this reduction causes a degradation of the so-called ‘plasticity reserve’ as the ratio between yield strength and UTS, affecting formability evaluation.
- During the experimentation, it was found that there was an average yield strength increase of 74 MPa as a result of strain rate, along with an average ultimate tensile strength increase of 30 MPa. Elongation decrease is greater than 10% in certain cases within the scope of deformation rates evaluated.
- When considering the position of the sample in the coil, it was found that batch C is the most sensitive to changes in yield strength values when samples are oriented at the sheet-rolling direction. The scatter of elongation values, however, was consistent across all batches.
- The results presented in this article indicate that the chemical composition of individual batches of the same material can significantly affect sheet deformability by altering its mechanical properties. Depending on the sample type, the difference in major strains between optimistic and pessimistic variants can range from 20.9% to 28.32%. Simultaneously, the difference between the minor strain points for pessimistic and optimistic variants can be as much as 26.2%.
- Analysis of the microstructure observed on the metallographic cut etched with NITAL 2% etchant revealed regular ferritic grains with an average grain size of 20 μm. They correspond to the expected size. For automotive sheets, it is stated that there should be 50–100 grains in the thickness of the material. Yellow, sharp-edged formations in the structure consisting of Al, Ti, C, and N were observed. From the metallographic analyses performed, it follows that with the increasing rate of deformation, the size of the deformation is localized to a smaller distance from the crack. Furthermore, deformation occurs mainly in the grains at a small depth below the surface. With increasing depth or distance from the crack, the amount of grain deformation decreases. The same observation applies to all analyzed stress states.
- The measured data can be used to refine material cards for numerical simulations and to achieve a more robust forming process. In numerical simulations, attention will be given not only to the initial conditions but also to the position of the material within the coil, the specific heat, and the supplier.

## Figures and Tables

**Table 1:** Mechanical properties of DX56D+Z100-M-C-O steel.

Yield Strength R_p0.2_, MPa	Tensile Strength R_m_, MPa	Elongation		Anisotropy		Strain-Hardening Exponent n_10–20/Ag_
		A_50_, %	A_80_, %	r_90/20_	r_m/20_	
140–180	270–330	≥40	≥39	≥1.9	≥1.6	≥0.20

**Table 2:** Chemical composition of DX56D+Z100-M-C-O steel (in wt.%).

C	Si	Mn	P	S	Al	Ti	Fe
≤0.06	≤0.50	≤0.40	≤0.025	≤0.025	≥0.010	≤0.30	balance

**Table 3:** Strain values for batch B for a test speed of 2 mm/s.

BS			BC			BE			B Average		
Sample	ε_2_ (Minor)	ε_1_ (Major)	Sample	ε_2_ (Minor)	ε_1_ (Major)	Sample	ε_2_ (Minor)	ε_1_ (Major)	Sample	ε_2_ (Minor)	ε_1_ (Major)
G1	−0.373	0.727	G1	−0.354	0.675	G1	−0.375	0.727	G1	−0.368	0.710
G2	−0.096	0.494	G2	−0.095	0.488	G2	−0.104	0.537	G2	−0.098	0.506
G3	0.000	0.381	G3	0.000	0.364	G3	0.000	0.382	G3	0.000	0.376
G4	0.145	0.375	G4	0.137	0.421	G4	0.081	0.377	G4	0.121	0.391
G5	0.245	0.459	G5	0.237	0.457	G5	0.249	0.451	G5	0.244	0.456

**Table 4:** Strain values for batch B for a test speed of 17 mm/s.

BS			BC			BE			B average		
Batch B	ε_2_ (Minor)	ε_1_ (Major)	Batch B	ε_2_ (Minor)	ε_1_ (Major)	Batch B	ε_2_ (Minor)	ε_1_ (Major)	Batch B	ε_2_ (Minor)	ε_1_ (Major)
G1	−0.324	0.600	G1	−0.327	0.613	G1	−0.333	0.621	G1	−0.328	0.612
G2	−0.085	0.435	G2	−0.080	0.412	G2	−0.075	0.403	G2	−0.080	0.417
G3	0.000	0.328	G3	0.000	0.336	G3	0.000	0.334	G3	0.000	0.333
G4	0.066	0.343	G4	0.078	0.332	G4	0.069	0.359	G4	0.071	0.345
G5	0.146	0.373	G5	0.170	0.372	G5	0.219	0.380	G5	0.178	0.375

**Table 5:** Images of crack region for batch B.

Test Speed	Crack Region Under 200× Magnification [50 µm]		
	Uniaxial Stress State	Plane Stress State	Biaxial Stress State
2 mm/s	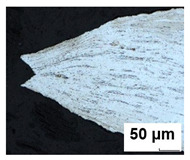	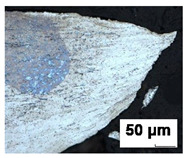	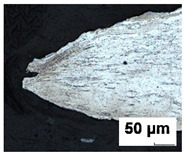
17 mm/s	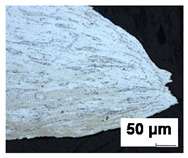	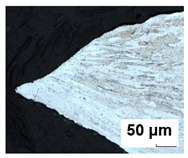	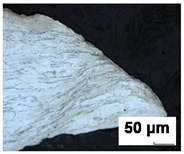

## Data Availability

The original contributions presented in this study are included in the article. Further inquiries can be directed to the corresponding author.
